# Evaluation of Deformable Image Registration for Three-Dimensional Temporal Subtraction of Chest Computed Tomography Images

**DOI:** 10.1155/2017/3457189

**Published:** 2017-10-12

**Authors:** Ping Yan, Yoshie Kodera, Kazuhiro Shimamoto

**Affiliations:** Department of Radiological and Medical Laboratory Sciences, Graduate School of Medicine, Nagoya University, 1-1-20 Daiko-Minami, Higashi-ku, Nagoya 461-8673, Japan

## Abstract

**Purpose:**

To perform lung image registration for reducing misregistration artifacts on three-dimensional (3D) temporal subtraction of chest computed tomography (CT) images, in order to enhance temporal changes in lung lesions and evaluate these changes after deformable image registration (DIR).

**Methods:**

In 10 cases, mutual information (MI) lung mask affine mapping combined with cross-correlation (CC) lung diffeomorphic mapping was used to implement lung volume registration. With advanced normalization tools (ANTs), we used greedy symmetric normalization (greedy SyN) as a transformation model, which involved MI-CC-SyN implementation. The resulting displacement fields were applied to warp the previous (moving) image, which was subsequently subtracted from the current (fixed) image to obtain the lung subtraction image.

**Results:**

The average minimum and maximum log-Jacobians were 0.31 and 3.74, respectively. When considering 3D landmark distance, the root-mean-square error changed from an average of 20.82 mm for *P*_fixed_ to *P*_moving_ to 0.5 mm for *P*_warped_ to *P*_fixed_. Clear shadows were observed as enhanced lung nodules and lesions in subtraction images. The lesion shadows showed lesion shrinkage changes over time. Lesion tissue morphology was maintained after DIR.

**Conclusions:**

DIR (greedy SyN) effectively and accurately enhanced temporal changes in chest CT images and decreased misregistration artifacts in temporal subtraction images.

## 1. Introduction

Deformable image registration (DIR) is a fundamental task in medical image processing and has many applications. One of the applications is the alignment of chest computed tomography (CT) images from the same subject and, in particular, of the lung and its internal structures for investigations, such as assessment of temporal structural changes or anatomical changes [1]. The applications of the information obtained from three-dimensional (3D) images have been rapidly increasing in the fields of diagnostics and surgical or radiotherapy planning. With the emergence of serial low-dose multidetector CT (MDCT) imaging for lung examination [2], the 3D temporal subtraction technique for chest CT has become a necessity for aiding in the diagnosis of conditions [3–5]. Temporal subtraction of serial volumes has the potential to efficiently identify areas with temporal changes, such as the lung, through approaches, such as visualization of tumor growth or shrinkage on subtraction images [6, 7]. However, because of the potential for misregistration between image volumes, direct subtraction typically does not achieve the desired results. Achieving accurate registration in repeated-interval chest CT scans is necessary to create temporal subtraction images for enhancing images of temporal changes in the lung, because successful registration will result in a perfect fit between lesions at two different time points. A subtraction image from alignment images is often used to evaluate the accuracy of a registration method. Dougherty et al. [6, 7] reported that serial images were aligned with at least a 0.95 correlation in five cases, and the subtraction image showed nodule growth involving an increase in size using the optical flow method.

Various image registration algorithms have been proposed for lung images in the literature. Moreover, “Evaluation of Methods for Pulmonary Image Registration 2010” [8] directly compared various different registration algorithms by applying each algorithm to the same set of pairs of thoracic CT images. In this study, we were interested in DIR involving diffeomorphic registration algorithms, which, by definition, preserve topology [9]. Preserving topology means that the structure in the deformed image maintains an adjacent relationship between the internal structures, connectivity is unchanged, tear or paste does not occur, no new structure appears, and the original structure does not disappear. A diffeomorphic transformation is defined as continuous mappings, with one-to-one correspondence between points in one image and points in the second image, and, for every position in one image, there is a signal corresponding position in the second image, with differentiability [10]. Diffeomorphic restriction is valid for a large number of problems in which the two images have the same structures and neighborhood relationships but the structures have different shapes [11]. However, diffeomorphic transformation is required for a geodesic connecting two images, *F* and *M*, in the space of diffeomorphic transformations, and the computational and memory costs are significant because of the dense-in-time velocity field calculations and requisite reintegration of the diffeomorphisms after each iterative update [12]. Avants et al. [13] introduced the greedy symmetric normalization (SyN) method as a lower-cost strategy, and the details on mapping images *F* to *M* and *M* to *F* by using the diffeomorphism *∅* and greedy optimization in diffeomorphic normalization have been previously described [9, 12, 13]. The study [9] describes and demonstrates two different diffeomorphic transformation methods for 3D image registration. The difference between a time-varying diffeomorphism (greedy SyN) and a diffeomorphism generated by exponential mapping has been described [14, 15], and comparison of both methods of lung image registration showed that the greedy SyN method was able to achieve top performance; however, the local lesion changes of the lung after diffeomorphic transformation were not reported. Diffeomorphic transformation may be used to identify areas where two image volumes differ topologically by analyzing the properties of the resulting transformation, such as when there is a problem of registration of an image with a large lesion to that of an image with a small lesion and when there is a problem of matching an image with a lesion to an image without a lesion [11].

In the present study, the aim was to perform lung volume DIR for the reduction in misregistration artifacts in temporal subtraction images to enhance temporal changes in lung lesions and evaluate the changes after DIR. DIR was implemented with MI lung mask affine mapping combined with CC lung diffeomorphic (greedy SyN) mapping using the advanced normalization tools (ANTs) software package (open-source software; http://picsl.upenn.edu/software/ants).

## 2. Materials and Methods

Our institutional review board approved this study (approval number is 14-315) and waived the informed consent requirement.

### 2.1. Patients

Chest CT images of 10 patients were obtained by a radiologist after a search of the radiology database. Each of the 10 patients had images taken at baseline and during follow-up (1–12 months apart). Each scan was obtained at full inspiration, and all images were obtained using an MDCT scanner (Aquilion 16; TOSHIBA Medical Systems, Japan). The images were acquired using the following parameters: volume size, 512 × 512 × slices; slice thickness, 5 mm; pixel spacing in the *X* and *Y* directions, range from 0.546 mm to 0.644 mm, with an average spacing of 0.595 mm.

### 2.2. Preprocessing

We maintained the continuity of chest CT images along the *z*-axis and obtained an accurate lung volume boundary. First, all image data with additional image slices in the gray-level lung volume were interpolated to obtain a resolution closer to isotropic resolution using subresample volume programs combining B-spline interpolation. Then, a binary lung mask was extracted using a shape detection level set filter combining a chamfer distance function, and the cavities were filled using a voting binary iterative hole-filling filter. Final, the lung tissue and lung mask volumes in paired temporal chest CT scans from the 10 patients were segmented. All of the processing approaches were performed using programs from the Insight Segmentation and Registration Toolkit (ITK) (https://www.itk.org).

### 2.3. Image Registration

The schema of DIR is based upon the following three principal components: (1) the transformation model (affine and greedy SyN); (2) similarity measures (MI and CC); and (3) the optimization strategy (multiresolution optimization parameters for both affine and greedy SyN).

Generally, for efficient registration of volumes, an affine registration is first used to capture global transformation. Thus, the affine transformation is optimized with respect to translation, rotation, scaling, and shearing with a mixture of 12 parameters.

The original diffeomorphic registration has symmetry properties required for a geodesic connecting two images,* F *and* M*, in the space of diffeomorphic transformations and guarantees symmetry regardless of the chosen similarity measures, such as cross-correlation (CC) [12]. However, the computational and memory costs are significant. The greedy SyN was developed as a lower-cost strategy, and its details can be obtained from the above-mentioned studies.

The basis of the ANTs MI function is the joint histogram of the images *F* and* M*, which is constructed by locating a joint intensity value at each position and then incrementing the nearest neighbor bin within the joint histogram. Implementation of MI and its gradient enables construction of an image-based joint histogram from which marginal distributions can be derived [13]. For lung CT imaging, the image pair is usually from two breathing phases, and the local density changes are linearly reflected in the intensity changes. In such a case, the invariance of CC to linear intensity change makes it a suitable similarity function [9]. ANTs compute CC in a neighborhood around each voxel to accommodate the inhomogeneity of density changes throughout the whole lung. The local CC is integrated over the lung volume as overall similarity in diffeomorphic transformation. The details of CC implementation have been previously described [9, 12, 16].

We performed greedy SyN mapping driven by CC and initialized by MI with an affine transformation. A four-level image pyramid was used to compute MI, and a five-level image pyramid was used to compute CC. These constituted the MI-CC-SyN implementation. In the affine transformation, lung masks were used as input images. A four-level image pyramid was used to compute MI, and optimization was performed at a maximum of 1000 iterations at the first coarsest level, 500 iterations at the next coarsest level, 250 iterations at the third coarsest level, and 100 iterations at full resolution. In the greedy SyN transformation, gray-level lung volumes were used as input images. Additionally, a five-level image pyramid was used to compute CC, and optimization was performed, with 100 iterations used at the first and second coarsest levels, 70 iterations at the third coarsest level, 50 iterations at the fourth coarsest level, and 20 iterations at full resolution. The same set of parameters was used across all image pairs. The neighborhood radius for computing the CC was two voxels and the weight was 1. Obtained affine and deformation field mapping was used to warp moving images to corresponding fixed images with linear interpolations. Finally, subtraction images of lung volume were obtained by subtracting the warped moving volume images from the current (fixed) volume images. The Jacobian of the deformation field is very informative with regard to the local properties of the deformation field and is greater than zero for topology-preserving mapping [1]. Jacobian > 1 implied local expansion, Jacobian < 1 implied local shrinkage, and Jacobian = 1 implied no change [8]. The log-Jacobian of the deformation field provides a contrast image in which low contrast areas tend to correspond to local shrinking and high contrast areas tend to correspond to local growing lesions.

The process was fully automatic and registration was implemented in Release 2.01 of the ANTs software package. Processing was completed using a 3.50 GHz Intel® Core™ i7-3770K CPU. The image registration average runtime was 2 h with MI-CC-SyN implementation.

## 3. Results

### 3.1. Quantitative Evaluation: Landmark-Based Accuracy Evaluation

The accuracy of image registration implementation was evaluated with landmark feature points identified in the fixed and corresponding moving images and warped moving images by an expert, with manual marking using 3D slice 4.5.0 software (https://www.slicer.org). The feature points were selected at the start of the upper lungs because pulmonary landmarks typically feature vessel bifurcation and most of the feature points are marked in the bifurcation of blood vessels included in the lung periphery. A set of 300–600 points was used to manually mark *P*_fixed_ in the fixed image and *P*_moving_ in the corresponding moving image from each scan pair. Each point in the fixed image was then manually matched with the corresponding point in the warped moving image (*P*_warped_). The distances from *P*_fixed_ to *P*_moving_ and from *P*_warped_ to *P*_fixed_ were calculated in mm for each of the point pairs. The root-mean-square (RMS) value and the average displacement of landmark feature points were both used to evaluate the fiducial registration errors of *P*_fixed_ to *P*_moving_ and *P*_warped_ to *P*_fixed_.  Table 1 shows the RMS errors and three average displacements of landmark feature points of *P*_fixed_ to *P*_moving_, for the 10 cases. The RMS error is related to the average 3D distance (average, 20.8 mm; range, 4.95 to 92.02 mm). The average displacement in each of the following three orthogonal directions: right-left (RL; average, 0.6 mm; range, −54.79 to 42.36 mm); anterior-posterior (AP; average, −8.8 mm; range, −42.52 to 10.92 mm); and superior-inferior (SI; average, 3.98 mm; range, −17.72 to 42.3 mm) are also shown before DIR.  Table 2 shows RMS errors and three average displacements of the landmark feature points of *P*_warped_ to *P*_fixed_. The RMS error is related to the average 3D distance (average, 0.5 mm; range, 0.42 to 0.68 mm). The average displacement in each of the following orthogonal directions: RL (average, 0.07 mm; range, −0.09 to 0.25 mm); AP (average, 0.03 mm; range, −0.15 to 0.25 mm); and SI (average, 0.06 mm; range, −0.07 to 0.21 mm), which are also shown after DIR.

### 3.2. Minimum and Maximum Jacobian (Logarithmic)

We investigated the minimum and maximum log-Jacobians of the MI-CC-SyN implementation among the 10 cases. The mean Jacobian minimum was 0.31, and the mean Jacobian maximum was 3.74 (Table 3).

### 3.3. Visual Inspection for Accuracy

**Figure 1:**
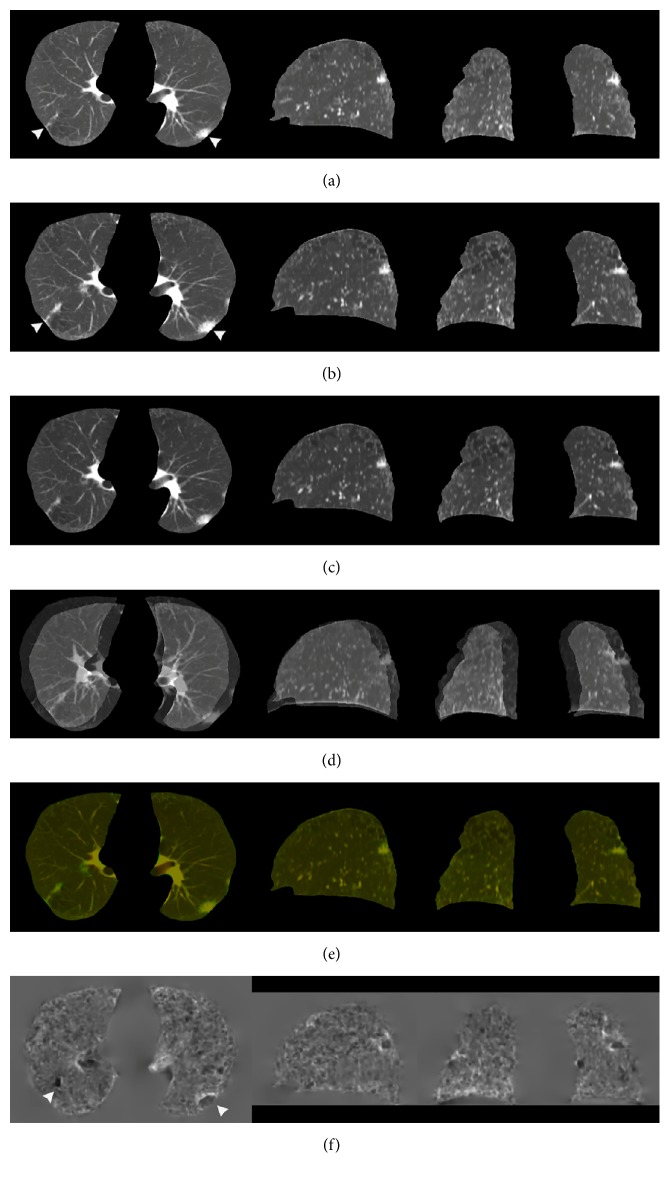
Sample output of deformable image registration. The columns from left to right are the axial, sagittal, and coronal planes, respectively. The white arrows indicate the left lung lesion and the right lung latent lesion. (a) Moving images (previous images) in the axial plane. The lengths of the long and short diameters of the left lung lesion are 33 and 20 mm, respectively. (b) Fixed images (current images, interval of 4 months 6 days). In the axial plane, the long and short diameters of the left lung lesion are 40 and 18.5 mm, respectively. (c) Warped moving images. The long and short diameters of the left lung lesion are 33 and 20 mm, respectively. (d) Overlapping of moving and fixed images (showed misregistration). The position of the lung surface shows a clear difference between moving and fixed volumes. (e) Overlapping of the warped moving and fixed images (well-matched). Red indicates the warped moving image and green indicates the fixed image. (f) Log-Jacobian image of the deformation field.

**Figure 2:**
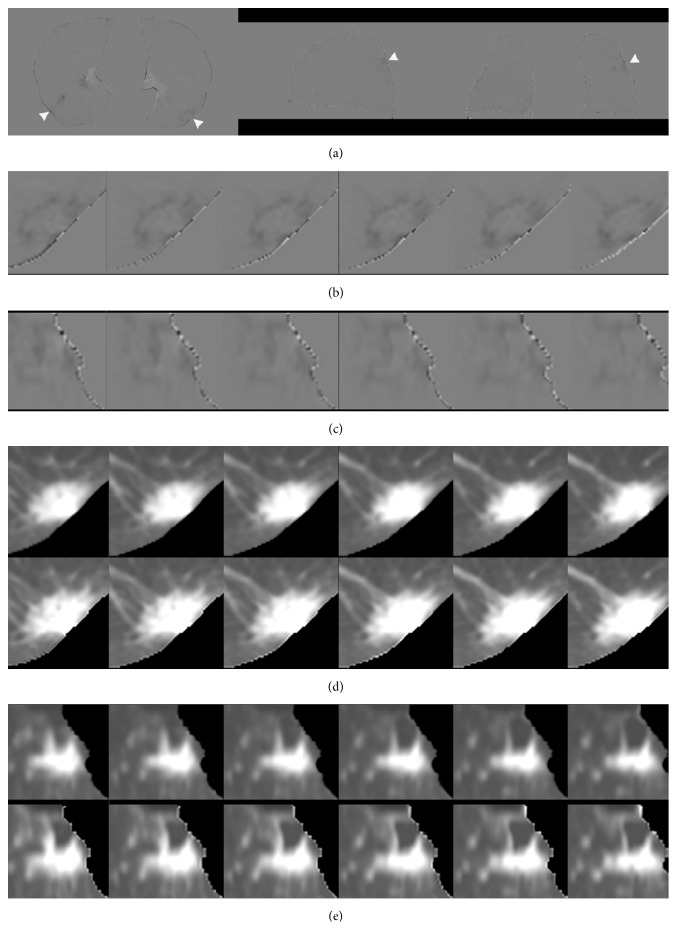
Sample output of the subtraction process and volume change tracker of Figure 1 after deformable image registration. The white arrows indicate the left lung lesion and right lung latent lesion. (a) Subtraction images were obtained by subtracting the warped moving images from the fixed images. (b), (c) Local enlarged subtraction images of the left lung lesion with six successive slices in the axial and coronal planes (showed clear enhanced shadows). (d), (e) Local enlarged images of lesion tissue with six successive slices and detailed comparisons of the morphological changes. In (d) and (e), the above row shows lesions in the warped moving images, while the below row shows lesions in the fixed images. No growth or shrinkage is noted. Perfectly fitted lesions are show in the two planes.

**Figure 3:**
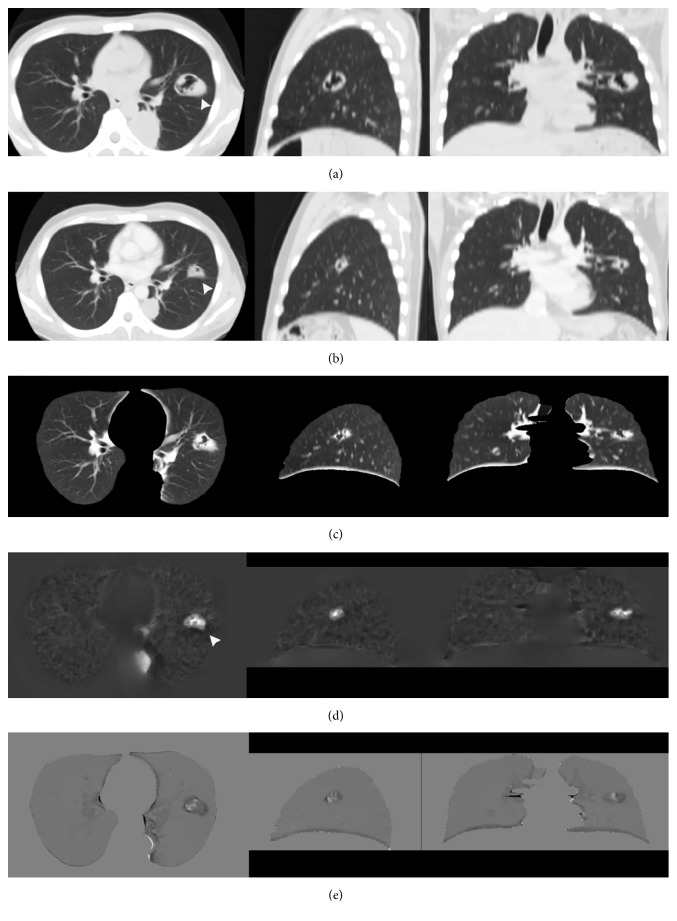
Comparison of the alignments of large lung lesions after DIR. Columns from left to right are the axial, sagittal, and coronal planes. The white arrows indicate lesions in the left lung. (a) Moving images (long and short diameters = 71 and 52 mm, resp.). (b) Fixed images (interval of 3 months 20 days; long and short diameters = 49 and 31.5 mm, resp.). (c) Warped moving images (long and short diameters = 55 and 36.8 mm, resp.). (d) Log-Jacobian image of the deformation field. (e) Subtraction images.

**Figure 4:**
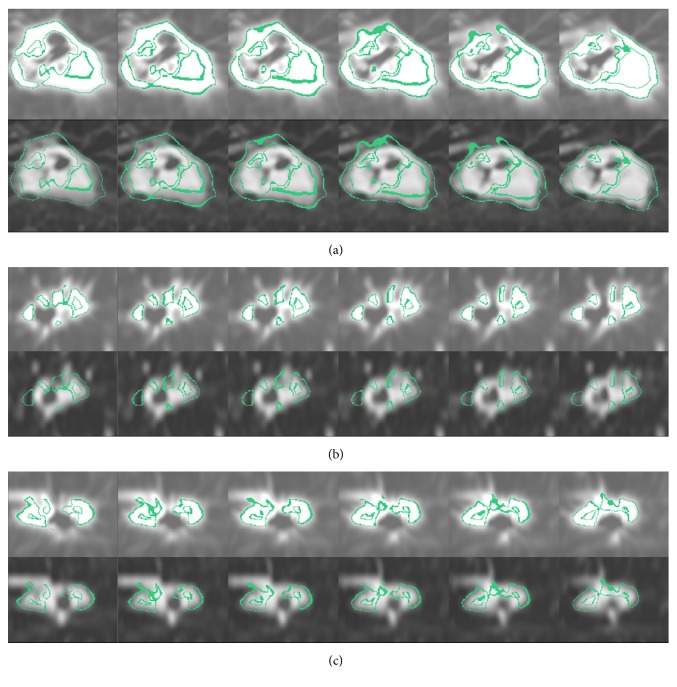
Volume change tracker for Figure 3 showing that lesion shrinkage changes with six successive slices in the three planes. The regions around the green lines show changes in warped moving and fixed images. (a), (b), and (c) Lesion in the axial, sagittal, and coronal planes. The above row shows the lesion in warped moving images, while the below row shows the lesion in fixed images. Clear shrinkage of the lesion is noted in the fixed image (0.65%) when compared with that in the warped moving image.

**Figure 5:**
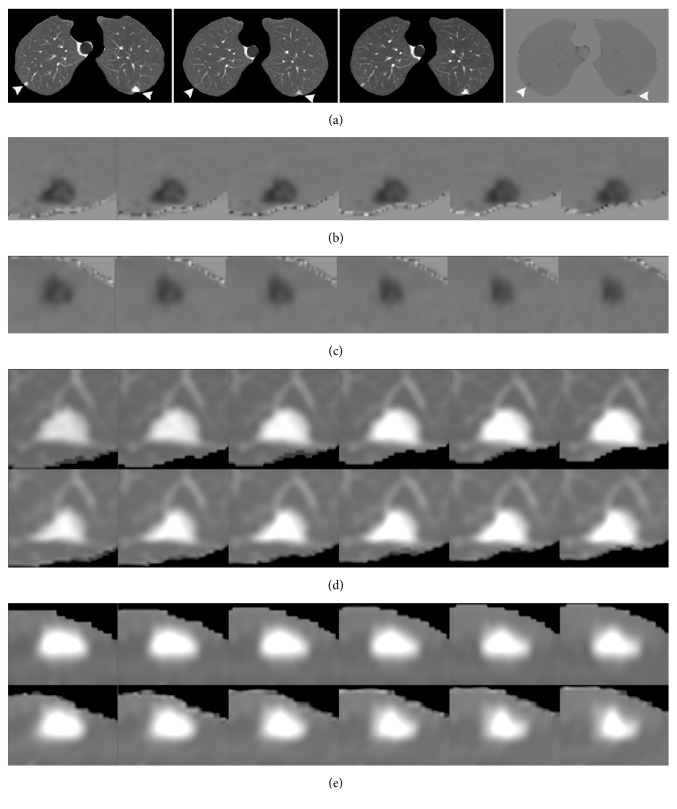
Comparison of the alignments of small metastatic nodules in the upper lung after deformable image registration. White arrows indicate small metastatic nodules in the left upper lung and subtle latent lesions in the right upper lung. (a) Columns from left to right are a moving image (left upper lung nodule diameter is 14 mm), fixed image (left upper lung nodule diameter is 10 mm), warped moving image (left upper lung nodule diameter is 13.6 mm), and subtraction image. (b), (c) Local enlarged subtraction images of the left upper lung nodule with six successive slices in the axial and coronal planes. Clear enhanced shadows are seen in the subtraction images. (d), (e) Local enlarged images of nodule with six successive slices and detailed comparisons of the morphological changes. In (d) and (e), the above row shows the nodule in moving images, while the below row shows the nodule in warped moving images. The nodule in the warped moving image retained nodular morphology when compared to that in the moving image.

Figures 1 and 2 show the sample output of DIR, subtraction process, and volume change tracker, respectively. The moving image (Figure 1(a), previous image) was registered to the fixed image (Figure 1(b), current image) to produce a warped moving image (Figure 1(c)). Results were qualitatively analyzed by comparing the difference in overlapping of original images (Figure 1(d)) with the difference in overlapping between warped moving and fixed images (Figure 1(e)).  Figure 1(f) (log-Jacobian image) illustrates the deformation field attained in DIR.  Figure 2 illustrates the subtraction image obtained by subtracting the warped moving images from the fixed images and volume change tracker for Figure 1.  Figure 3 shows comparison of large lung lesions after DIR.  Figure 4 shows volume change tracker for Figure 3.  Figure 5 shows comparison of the alignment of small metastatic nodules in the upper lung after DIR.

In Figure 1, a right lung latent lesion was identified. The problem involved the registration of the image without a latent lesion (Figure 1(a)) to that with a lesion (Figure 1(b)). After DIR, we found that the latent lesion was clearly noted in the warped moving images (Figure 1(c)) and corresponding subtraction images (Figure 2(a)). In Figure 1(f), a low-density Jacobian region was noted, and it indicated the local deformation field of the right lung latent lesion.

With regard to the accuracy of visual evaluation, on comparing the difference in overlapping among original images with the difference in overlapping between warped moving and fixed images, we found that the later overlapping images showed exactly aligned lung volumes, with a well-matched lung boundary in the periphery of the lung after DIR.

In subtraction images, the local enlarged subtraction images for lesions and nodules illustrate exactly aligned lung volumes (Figures 2(a)–2(c) and 5(b)–5(c)). There were decreased misregistration artifacts, and the shadows of the lesions and nodules showed clear enhancement upon visual inspection.

A volume change tracker compares the details of morphological changes in lesion tissue between warped moving and fixed images. In Figures 2(d)–2(e), there is a perfect fit of lesions in the axial and coronal planes, and no growth or shrinkage is noted. The size assessments of the lesion show no size change after DIR.

Figure 3 illustrates large lesion shrinkage changes. We found that the lesion size was lower in the warped moving image than in the original moving image (long diameter, 55 mm; short diameter, 36.8 mm versus long diameter, 71 mm; short diameter, 52 mm). However, the lesion size was greater in the warped moving image than in the fixed image (long diameter, 55 mm; short diameter, 36.8 mm versus long diameter, 49 mm; short diameter, 31.5 mm). In addition, we found that the lesion in the warped moving image had greater morphological characteristics than the lesion in the fixed image. In the log-Jacobian image (Figure 3(d)), a high-density region was noted, and it illustrated the local deformation field of the large lesion. The dark shrinkage shadows and residual lesions are clearly shown in the subtraction images (Figure 3). The volume change tracker (Figures 4(a)–4(c)) indicated that the lesion in the fixed image showed shrinkage (0.65%) compared with that in the warped moving image. Moreover, the lesion appeared in 25 successive slices of the moving image and 16 successive slices of the fixed image that confirmed shrinkage of the DIR.

In Figure 5, a right upper lung subtle latent lesion was noted. The problem involved registration of the image with a latent lesion (Figure 5(a), moving image; left image) to that without a lesion (Figure 5(a), fixed image; middle image). After DIR, we found that the latent lesion was clearly noted in the warped moving images (Figure 5(a); middle image) and corresponding subtraction images (Figure 5(a); right image). Furthermore, in Figure 5, a left upper lung metastatic nodule was identified. The problem involved registration of the image with a large nodule to that with a small nodule. After DIR, we found that the nodule in the warped moving images had greater morphological characteristics than the nodule in the fixed images. The volume change tracker indicated that the nodule in fixed images showed shrinkage when compared with that in warped moving images.

## 4. Discussion

Various image registration algorithms have been surveyed for medical image registration in the literature [1, 17–22]. However, currently, translating the results of image registration research to clinical practice is urgently needed [17]. The temporal subtraction of aligned volumes can simply and efficiently identify areas of chest interval changes; however, a successful registration to reduce misregistration artifacts is essential. Currently, temporal subtraction is mainly used to detect a new nodule or nodule growth [23]. Itai et al. [5] developed a voxel-matching technique for substantial reduction of subtraction artifacts in temporal subtraction images from thoracic MDCT. This registration method involved global image matching (2D CC), 3D local elastic matching, and 3D nonlinear image warping (3D voxel matching). However, this technique tends to reduce the size of large lesions because it removes the low-level background of subtraction image, and it appears difficult to find the low-density shadow. In this study, low-density shadows in subtraction images presented local volume shrinkage changes in the lesion over time. Such changes can help physicians detect and analyze pathological changes and the effects of a drug or therapy.

Topology preservation is fundamental for making comparisons between objects in the natural world. Song et al. described the use of diffeomorphic transformation models in the EMPIRE10 challenge. They found that most fissures and lung boundaries were aligned well, with an error close to zero, and that there were almost no singularities in the deformation fields owing to the theoretical properties of diffeomorphic transformation models [9]. Avants et al. reported the best diffeomorphic results in a brain study involving the initialization of MI-based affine registration [12]. They found that MI affine mapping combined with CC diffeomorphic mapping provided the best cortical labeling results. Thus, in ANTs, MI-based affine mapping provides the best initialization of deformable registration. In addition, CC is considered a more suitable similar function for lung CT images. In this study, we obtained lung subtraction images by applying MI-CC-SyN image registration method using ANTs for chest CT images in 10 cases. Paired landmark feature points were using to qualitatively evaluate the accuracy of registration. The RMS error in the average 3D distance of landmark feature points in the 10 cases was 20.82 mm before registration, and it greatly reduced to an average of 0.5 mm after registration. The resulting subtraction images showed excellent registration results for lung volume and lung boundary in the periphery of the lung with MI-CC-SyN implementation. The DIR may minimize the difference between lesions at various time points, thereby concealing lesion growth or shrinkage [24]. We also investigated local changes in the lesions after MI-CC-SyN implementation; the results showed that the size and morphological characteristics of the lesions or nodules were maintained and the subtle latent lesions were matched. With regard to a large lesion (Figure 3), the lesion lumen inhomogeneous density appeared in the moving image, whereas the lesion size significantly reduced and the cavity significantly narrowed in the fixed image. After MI-CC-SyN implementation, internal morphological characteristics of the lesion were maintained in the warped moving image. The Jacobian provided contrast images related to the volume variation of each lesion. The contrast areas corresponded to the maximum shrinkage as in Figure 1(f) (right lung, white arrow), which shows lesion shrinkage in the moving image, and the maximum expansion as in Figure 3(d) (white arrow), which shows lesion expansion in the moving image. The subtraction image clearly shows changes. These findings imply that the underlying topology was preserved. The original structure of the lesion or nodule is not lost after MI-CC-SyN implementation, indicating that the MI-CC-SyN implementation of ANTs can be used to identify areas where two image volumes differ topologically. The limitation of diffeomorphic transformation has been shown to be invalid when registering images before and after surgery [11].

The study of a temporal series of medical images can help physicians in patient follow-up. In some diseases, lesions, nodules, or anatomical structures vary in size, position, composition, and so forth, over time either because of a natural pathological process or because of the effect of a drug or therapy [25]. Thus, it is important to detect regions with apparent local volume variations. Therefore, after DIR, the temporal and intensity changes of the lesion tissue should be preserved in the subtraction image. Our results indicate that MI-CC-SyN implementation is particularly well suited as a registration algorithm for achieving accurate registration in repeated-interval chest CT images that can be used to create temporal subtraction images for enhancing images of temporal changes in lung lesions.

The main limitation of this study was the use of only 10 cases. We did not extract the main airways in lung segmentation, and there was no comparison of other registration methods. In the future, a large data set is needed to validate our findings, and attempts should be made to transfer the results of image registration research to clinical practice. The minimization of misregistration artifacts is important for accurate temporal subtraction image processing because misregistration artifacts may result in failure to detect interval changes.

## 5. Conclusions

To enhance the ability to detect interval changes in subtle lung lesions by using a subtraction image, we used MI lung mask affine mapping combined with CC lung tissue greedy SyN mapping to accurately register temporal serial chest CT images. The obtained subtraction images clearly showed significantly decreased misregistration artifacts and enhanced lesions and nodules in the lung volume. Furthermore, the lesions maintained original morphology after registration. These results indicate that MI lung mask affine mapping combined with CC lung tissue diffeomorphic mapping (greedy SyN) in ANTs is useful for temporal subtraction of 3D chest CT images. Thus, 3D temporal subtraction images could be beneficial for enhancing images of temporal changes in lung lesions and may improve a radiologist's ability to detect lung cancer at an early stage.

## Figures and Tables

**Table 1:** The root-mean-square error of fiducial registration with regard to the 3D average distance and the average displacement of landmark feature points in the following three directions before DIR in the 10 cases: right-left (RL), anterior-posterior (AP), and superior-inferior (SI).

Cases	1	2	3	4	5	6	7	8	9	10	Avg.
3D RMS (mm)	19.06	5.04	6.35	4.95	92.02	25.67	5.43	17.16	8.43	24.04	20.82
RL (mm)	4.57	10.62	23.17	−13.31	42.36	3.93	17.77	−54.79	−48.07	20.04	0.63
AP (mm)	1.16	−4.81	5.33	−8.95	−34.68	5.22	0.66	−42.54	−20.41	10.92	−8.81
SI (mm)	3.01	3.55	20.85	−17.72	6.52	−14.91	16.92	−9.52	42.26	−11.18	3.98

**Table 2:** The root-mean-square error of fiducial registration with regard to the 3D average distance and the average displacement of landmark feature points in the following three directions after DIR in the 10 cases: right-left (RL), anterior-posterior (AP), and superior-inferior (SI).

Cases	1	2	3	4	5	6	7	8	9	10	Avg.
3D RMS (mm)	0.68	0.55	0.63	0.42	0.49	0.54	0.59	0.59	0.51	0.44	0.54
RL (mm)	−0.05	−0.05	0.11	−0.04	0.13	017	0.09	0.14	0.25	−0.09	0.06
AP (mm)	−0.13	0.16	−0.01	0.02	0.07	−0.15	0.25	0.06	0.05	−0.03	0.02
SI (mm)	−0.07	−0.02	0.12	0.02	0.02	0.15	0.2	0.09	0.21	−0.07	0.064

**Table 3:** The Min and Max Jacobians according to MI-CC-SyN (Log).

Case	1	2	3	4	5	6	7	8	9	10	Avg.
Min	0.30	0.40	0.33	0.26	0.33	0.33	0.37	0.30	0.29	0.21	0.31
Max	2.86	5.55	5.96	2.62	3.04	2.64	2.20	3.57	4.37	2.73	3.74

MI, mutual information; CC, cross-correlation; SyN, symmetric normalization; Min, minimum; Max, maximum.
